# Mandarin-speaking children with different types of cochlear implant exhibit variations in the activation patterns of their central auditory processing

**DOI:** 10.3389/fnins.2024.1520415

**Published:** 2024-12-16

**Authors:** Xiang Mao, Ziyue Zhang, Yijing Yang, Yue Wang, Yu Chen, Wei Wang

**Affiliations:** ^1^Department of Otorhinolaryngology Head and Neck Surgery, Tianjin First Central Hospital, Tianjin, China; ^2^Institute of Otolaryngology of Tianjin, Tianjin, China; ^3^Key Laboratory of Auditory Speech and Balance Medicine, Tianjin, China; ^4^Key Medical Discipline of Tianjin (Otolaryngology), Tianjin, China; ^5^Otolaryngology Clinical Quality Control Centre, Tianjin, China

**Keywords:** Mandarin pronunciation multifeature paradigm, source localization analysis, bilateral cochlear implantation, unilateral cochlear implantation, bimodal stimulation

## Abstract

**Background:**

Cochlear implants (CIs) have the potential to facilitate auditory restoration in deaf children and contribute to the maturation of the auditory cortex. The type of CI may impact hearing rehabilitation in children with CI. We aimed to study central auditory processing activation patterns during speech perception in Mandarin-speaking pediatric CI recipients with different device characteristics.

**Methods:**

We developed and implemented a multifeature paradigm for Mandarin pronunciation to capture mismatch negativity (MMN) responses in pediatric CI recipients, analyzed the cortical processing sources of MMN responses elicited by different stimuli, and identified significant differences in the frontal cerebral cortex activation between different types of CIs located in the corresponding brain regions according to the Anatomical Automatic Labeling (AAL) brain template. The clinical characteristics, aided hearing threshold (AHT), and speech perception accuracy (SPA) of these children were also recorded.

**Results:**

This study involved 32 pediatric CI recipients, with 12 (37.5%) receiving unilateral implants, 10 (31.3%) receiving bilateral implants, and 10 (31.3%) receiving bimodal stimulation. The cortical areas involved in the MMN response to various Mandarin pronunciation stimuli showed the greatest activity in the prefrontal lobe. In children with bimodal stimulation, there was noticeable activation in prefrontal cortical areas. Children with unilateral and bilateral implants also showed activation of the prefrontal cortex, but the activation strength was relatively reduced. The activation of cortical areas did not consistently appear stronger in children with bilateral implants than in those with unilateral implants. Consonant and intensity stimuli showed greater activation, whereas duration and vowel stimuli showed weaker activation. Significant differences in frontal cerebral cortex activation between different types of CIs were predominantly observed in the superior frontal gyrus.

**Conclusion:**

Bimodal stimulation should be considered whenever possible to maximize auditory benefits. For deaf children without any residual hearing, bilateral implantation is the best choice. Unilateral implantation is not as detrimental as previously thought for deaf children. Early cochlear implantation, comprehensive auditory training, and better adaptation to CI devices can efficiently compensate for unilateral hearing limitations.

## 1 Introduction

Hearing impairment (HI) is a common congenital disability. HI affects approximately 1 to 3% of newborns, with severe to profound cases accounting for approximately 1% of the population (Kenna et al., 2007). Childhood is a crucial time for developing language skills, and HI can cause delays in language development, which may subsequently impact cognitive, psychological, and social abilities (Lieu et al., 2020). Cochlear implants (CI) can assist in restoring hearing and fostering communication skills for deaf children by using electrical stimulation to activate the auditory nerve and promote the development of brain cortices. It has been reported that over 25,000 deaf children in mainland China have received CIs. Some provinces’ basic medical insurance scheme covers unilateral implants for children (Li et al., 2017).

However, not all children with CIs achieve optimal rehabilitation outcomes, and numerous studies have shown that these children’s language and neurocognitive outcomes vary significantly among individuals (Tamati et al., 2022). The observed variations in clinical outcomes may be attributed to several factors, including the age of implantation (Kral, 2007), duration of device usage (Calmels et al., 2004), onset of hearing loss (Dowell et al., 2002), residual hearing (Zwolan et al., 1997), etc. Among them, the types of CIs, e.g., unilateral or bilateral implants or bimodal stimulation, should also be considered.

The evidence suggests that bilateral implants and bimodal stimulation are more effective than unilateral implants, which has been extensively demonstrated in Western countries. For instance, the pediatric population with bilateral implants demonstrates better speech perception in quiet and noisy environments and enhanced sound localization compared with those with unilateral implants (Sparreboom et al., 2010). Incorporating a hearing aid (i.e., bimodal stimulation) contralaterally allows unilateral CI users to utilize residual auditory function in the non-implanted ear, thereby alleviating auditory deprivation and promoting binaural integration, demonstrating the advantages of bimodal stimulation in sound localization, music perception, and speech understanding (Beijen et al., 2008; Ching et al., 2007).

With improvements in socioeconomics and increasing recognition of the significance of binaural hearing, bilateral implant or bimodal stimulation has also been an emerging focus for researchers in mainland China (Long et al., 2018; Chen and Wong, 2020). In contrast to English, Mandarin is characterized by its tonal nature, featuring four distinct lexical tones that convey meaning at the level of individual syllables (Fu et al., 1998). Consequently, the auditory perception characteristics of Mandarin-speaking children with CIs are expected to differ from those of English-speaking children. A previous study indicated that children with bimodal stimulation demonstrated significantly better performance on the Meaningful Auditory Integration Scale (MAIS) and the Categories of Auditory Performance Questionnaire (CAPQ). However, another study indicated that, at 24 months post cochlear implantation, children with bilateral implants did not demonstrate better performance in the LittleEARS^®^ Auditory Questionnaire (LEAQ) compared with those with unilateral implants, as both groups had a ceiling effect. While evidence supporting the efficacy of binaural hearing in enhancing speech perception continues to accumulate, further research is imperative to substantiate the benefits of bimodal and bilateral implantation in Mandarin-speaking children with CIs (Gao et al., 2021).

Electroencephalography (EEG) has provided a powerful and accessible neuroimaging tool for investigating human brain physiology and cognition (Biasiucci et al., 2019). It has been possible to use neurophysiological methods to explore perceptual and cognitive deficits in children with CIs, obtaining quantitative data on brain activity while avoiding the biases inherent in administering face-to-face psychometric tests. The event-related potential (ERP) offers an objective and time-sensitive measurement of central auditory processing and can be recorded via EEG. In recent decades, Gordon’s EEG-based studies have revealed variations in central auditory processing activation patterns among pediatric CI recipients by capturing the electrically evoked auditory brainstem response (EABR), the P1, or N1 components. They indicated that unilateral implants may disrupt typical bilateral auditory pathways, leading to an unusually large asymmetry in auditory brainstem activity (Gordon et al., 2007; Gordon et al., 2008). The exclusive input from a single CI without intervention for deafness in the contralateral ear enhances neural pathways on the stimulated side while impeding maturation or inducing degenerative changes or reorganization of developing pathways from the deprived ear (Gordon et al., 2008). Bilateral implants thus protect the auditory cortices from unilaterally driven changes to sustain normal cortical lateralization and stimulus preference (Gordon et al., 2013; Polonenko et al., 2018).

Informed by Gordon’s findings, we aimed to investigate central auditory processing activation patterns during speech perception in Mandarin-speaking pediatric CI recipients with varying device characteristics. It is widely recognized that specific subprocesses play a role in the function of speech perception during the early preattentive stage (the automatic process outside conscious attention) (Pulvermüller and Shtyrov, 2006). This stage has been investigated by analyzing the mismatch negativity (MMN) responses of ERPs (Sorokin et al., 2010). MMN responses can be elicited by presenting a sequence of identical auditory stimuli with intermittent variations, elucidating how the auditory cortex responds to perturbations in regular patterns (Näätänen et al., 2007). Source localization revealed a larger MMN in the frontotemporal cortex from 150 to 250 ms for proficient language users, highlighting the important role of the frontal cortex in language perception (Pulvermüller and Shtyrov, 2006; Pulvermüller and Shtyrov, 2009; Pulvermüller and Fadiga, 2010). The array of cortical sources revealed by the MMN in processing higher language functions far exceeds those associated with classic sensory components, such as the N1 response to basic acoustic stimuli (Pulvermüller et al., 2004). Consequently, we opted to utilize the MMN as a biomarker to investigate the activation patterns of central auditory processing during speech perception.

To optimize the measurement of the MMN responses to different Mandarin phonemic features, we designed and constructed a Mandarin pronunciation multifeature paradigm consisting of a standard stimulus and five different phonemic deviant stimuli (Mao et al., 2024). This paradigm enables the recording of MMN responses to various Mandarin pronunciation changes within a single session, providing a comprehensive understanding of auditory processing within a relatively brief timeframe (Näätänen et al., 2004). This feature makes it particularly advantageous for pediatric assessments. The reliability of the multifeature paradigm has been verified in several studies (Pakarinen et al., 2009; Niemitalo-Haapola et al., 2013). Furthermore, to precisely identify the cortical processing origins of the MMN response to diverse Mandarin pronunciation stimuli, EEG data were acquired using a 256-electrode high-density EEG system. Several studies have investigated the advantages of increasing the number of EEG electrodes, suggesting that increasing the electrode count can enhance source imaging and localization accuracy (Lantz et al., 2003; Väisänen and Multichannel, 2008; Hassan et al., 2014). Recent experimental evidence has demonstrated that increasing the number of electrodes up to 256 amplifies the spatial information content of the inverse cortical potential distribution derived from scalp potentials (Väisänen and Multichannel, 2008).

In summary, this study aims to fill the knowledge gap regarding the activation patterns of central auditory processing in Mandarin-speaking children with different types of CIs. Initially, we developed and implemented a multifeature paradigm for Mandarin pronunciation to capture MMN responses. We subsequently analyzed the cortical processing sources of MMN responses elicited by different stimuli. Finally, we identified differences in the activation of the frontal cerebral cortex among different types of CIs and located them in frontal brain regions corresponding to the Anatomical Automatic Labeling (AAL) brain template. Furthermore, we conducted a supplementary analysis of the clinical characteristics, aided hearing threshold (AHT), and speech perception accuracy (SPA) of these children. This study facilitates a deeper understanding of the variations in central auditory processing activation patterns among Mandarin-speaking children with diverse CIs.

## 2 Materials and methods

### 2.1 Study participants and questionnaire

Between June 2023 and January 2024, Mandarin-speaking pediatric recipients of CI admitted to Tianjin First Central Hospital’s Department of Otorhinolaryngology-Head and Neck Surgery were recruited if they met the criteria for cochlear implantation and had previously undergone the procedure at our institution (Cochlear Implant Work Guide, 2013). The inclusion criterion was an age younger than 18 years. According to the Work Guidelines for Cochlear Implantation in China, the age at cochlear implantation ranged from 1 to 6 years old, and the subjects had entered the stable rehabilitation period after cochlear implantation, within which the AHT was within the speech banana map. The exclusion criteria encompassed severe cochlear malformations, dysplasia of auditory nerves or neuropathy, epilepsy, and neurodevelopmental disorders such as autism or attention deficit hyperactivity disorder. Furthermore, the study excluded the data of pediatric recipients of CI who were uncooperative with medical personnel during AHT and SPA testing, as well as the EEG data of pediatric recipients of CI where the electroencephalographic physiological signals were disrupted by involuntary fidgeting of the subjects and abnormal artifacts of their cochlear devices.

Before conducting the assessments, the medical team provided detailed explanations of the study objectives, test procedures, data usage, and privacy safeguards to patients and their families. Informed consent was then obtained from either the participants or their guardians before data collection. The enrolled participants completed a comprehensive questionnaire on sociodemographic factors, the progression of hearing impairment, surgical intervention, and postoperative rehabilitation under the guidance of healthcare professionals. The questionnaire data collected via the online data collection software were directly transferred into a structured database.

### 2.2 Institutional review board statement

This study complied with the Declaration of Helsinki. The Medical Ethics Committee of Tianjin First Central Hospital approved the research protocol. The review number is 2020N114KY. All participants were comprehensively briefed on the study. Informed consent was obtained from all subjects involved in the study. Written informed consent was obtained from the patients to publish this paper.

### 2.3 AHT and SPA test

Prior to administering the AHT and SPA tests, the audiologist utilized an otoscope to examine the ear canal for cerumen and foreign objects, and assessed the functionality of the hearing aid (HA) or CI. Subsequently, adjustments were made to the parameters of the HA or CI based on the patient’s AHT test outcomes and feedback to achieve optimal stimulation levels. The AHT test employed a continuous warble tone sound signal, with testing conducted at frequencies of 500, 1,000, 2,000, and 4,000 Hz. The subjects were instructed to cooperate with the audiologist during the testing and indicate when they heard each sound by raising their hand. The SPA test was administered via the “Xin Ai Fei Yang” Chinese speech audiometric system developed by the PLA General Hospital (Luo et al., 2016), with a signal intensity set at 70 dB SPL. Monosyllabic words under quiet conditions were used as test stimuli in this study; each test comprised 25 syllables. The participants were required to immediately repeat monosyllabic words presented through a speaker. The scoring was based on the consonant accuracy, vowel accuracy, and tone recognition rate calculated by the system. Further details regarding the procedures for both the AHT and SPA tests can be found in our previous study (Mao et al., 2023).

### 2.4 Test procedure and Mandarin pronunciation multifeatured paradigm design

The multifeature paradigm for Mandarin pronunciation included a standard stimulus (50%) and five different types of deviants (10% each). The standard stimulus was a 70 dB SPL syllable/bā/lasting 200 ms with a rise and fall time of 50 ms. The deviants were as follows: deviant 1 involved a tone change from/bā/to/bà/; deviant 2 entailed a duration change from 200 ms to 300 ms; deviant 3 featured a vowel change from/bā/to/bī/; deviant 4 comprised a consonant change from/bā/to/pā/; and deviant 5 encompassed an intensity change from 70 dB to 77 dB. Professional male announcers recorded the Mandarin syllables in an acoustically insulated room, and they were normalized using Cool Edit Pro software (Syntrillium Software Corporation).

The standard and deviant stimuli were presented in a sequence comprising 5 standard stimuli and five deviant stimuli, with the entire stimulus sequence encompassing 120 cycles. Each interstimulus interval was randomly drawn from a range between 600 and 700 ms. The complete stimulus sequence comprised 600 presentations of standard stimuli, along with 120 presentations of each deviant stimulus. The timing and presentation of all the stimuli were precisely controlled by a computer operating E-Prime 3.0 software (Psychology Software Tools Corporation, USA). Further details regarding the test procedure can be found in a previous study (Mao et al., 2024).

### 2.5 EEG recording and data preprocessing

EEGs were recorded with EGI GES400 (EGI Corporation, USA) using a GSN-HydroCel™-257 saline electrode cap. During the experiment, Net Station Acquisition 5.4.3-R software was used to record the EEG data. The raw EEG data were preprocessed via the EEGLAB open-source toolbox for MATLAB (R2021a) software (MathWorks Inc., USA). The details of data preprocessing can be found in a previous study (Mao et al., 2024).

### 2.6 EEG data time-domain analysis

The time-domain analysis of EEG data was not the primary focus of this study. We conducted this analysis with the specific aim of identifying the time points at which MMN responses are elicited by different stimuli to provide a reference for source localization analysis. MMNs were defined as the waveforms resulting from deviant stimuli minus those from standard stimuli, which were then averaged across nine electrodes surrounding the Fz electrode. Time windows were determined separately for each Mandarin pronunciation stimulus based on the MMN waveforms by identifying latency at the lowest value within the 150–250 ms window following stimulus presentation. The mean amplitude topography was calculated using a ± 20 ms time window around the latency of the lowest value time points. The time points of the MMN responses were determined based on both the MMN waveform and the topography.

### 2.7 Source localization analysis

Source localization was conducted using the FieldTrip (version 20,220,819) toolbox for MATLAB (R2021a). The EGI GSN-HydroCel™-257 Sensor Net electrode locations were utilized in the source reconstruction for all the subjects, and these electrodes were coregistered to a volume conduction model. The volume conduction model was computed via the boundary element method based on T1 images of a standard brain from a 10-year-old child acquired with MRI equipment (Siemens 3.0 T MAGNETOM Trio Tim MRI equipment) at the Department of Radiology, Tianjin First Central Hospital (Hallez et al., 2007). This standard volume conduction model was applied to all participants. Minimum norm estimates (MNEs) were employed to solve the inverse problem of EEG source localization (Grech et al., 2008). The location of MMN response sources was determined as regions exhibiting 10*log_10_ (deviant stimulus power/standard stimulus power) values in the top 60% (McMackin et al., 2019). The mean sources of the MMN responses were calculated within a ± 20 ms time window around the identified latency of each Mandarin pronunciation stimulus trough based on the waveform and the topography by time-domain analysis. The activation power of MMN responses in each voxel subsequently coalesced into brain regions corresponding to the AAL brain template.

To investigate the differences in activated cortical regions of MMN responses to various Mandarin pronunciation stimuli among different types of CIs, a cluster-based random permutation procedure (a nonparametric statistical test) was performed (Maris and Oostenveld, 2007; Moreno et al., 2015). This method effectively controls the familywise error rate resulting from multiple statistical comparisons at the critical alpha level (Bullmore et al., 1999). First, an independent sample t test was conducted to identify significant voxels, and the T values were thresholded (*α* = 5%). All voxels with a T value exceeding the threshold were subsequently selected. Significant voxels were then clustered based on spatial adjacency, and a cluster-level test statistic was computed by summing all T values within each cluster to evaluate the statistical significance of individual clusters. The significance of each cluster-level statistic was assessed by comparing it to a permutation distribution derived from the data; cluster statistics falling in the top 5th percentile were considered significant (*α* = 5%). The permutation distribution was generated through 10,000 random permutations of the data. Due to the limited statistical power resulting from small sample sizes in independent sample t tests between different types of CIs (12 in unilateral implants, 10 in bilateral implants and 10 in bimodal stimulation), we aimed to augment the sample size by replicating the data fourfold. This procedure increases the statistical power and ensures the reliability of the statistical findings, thereby ensuring the detection of activated cortical regions associated with MMN responses across different types of CIs with statistical significance, despite the limited sample size. The xjView toolbox^1^ for MATLAB (R2021a) was utilized to identify voxels demonstrating statistical significance to the cortex regions corresponding to the AAL brain template.

### 2.8 Statistical analysis

The AHT and SPA test results were entered into Epidata 3.1 software (Epidata Association, Inc.) and stored in the database. The frequencies of 500, 1,000, and 2000 Hz contributed 70% of the language intelligibility. In 1997, the World Health Organization (WHO) established a classification standard that included an additional hearing threshold of 4,000 Hz to address higher-frequency hearing loss in deaf individuals. Therefore, this study utilized the average AHT across these four frequencies as the AHT for each ear (in dB). We specifically examined superior AHT and SPA outcomes from both ears in pediatric patients with bilateral implants and bimodal stimulation for further analysis. One-way analysis of variance (ANOVA) was employed to compare differences in characteristics among pediatric CI recipients and the activation power of MMN responses across various frontal cortex brain regions associated with different types of CIs. Bonferroni correction was used for *post hoc* analyses. IBM SPSS Statistics 20.0 (IBM, Inc.) was used for the statistical analysis, and *p* < 0.05 was considered a statistical significance.

## 3 Results

### 3.1 Clinical characteristics of the subjects

This study included a total of 32 pediatric CI recipients. The mean age of the children was 9.4 ± 4.1 years. Sensorineural hearing loss (SNHL) (43.8%) and large vestibular aqueduct syndrome (LVAS) with SNHL (43.8%) were the predominant etiologies of HI. Unilateral implants were utilized in 12 children (37.5%), bilateral implants were utilized in 10 children (31.3%), and bimodal stimulation was employed in 10 cases (31.3%). Other clinical characteristics of the subjects are shown in Table 1. The average duration of auditory deprivation was 22.0 ± 23.0 months, the mean age at cochlear implantation was 2.5 ± 2.1 years, and the mean duration of cochlear implantation was 82.2 ± 49.7 months.

**Table 1:** Clinical characteristics of 32 Mandarin-speaking pediatric CI recipients.

	*n*	%
Gender		
Men	11	34.4
Women	21	65.6
The types of CIs		
Unilateral implants	12	37.5
Bilateral implants	10	31.3
Bimodal stimulation	10	31.3
The reasons for deafness		
SNHL+LVAS/Mondini	14	43.8
SNHL	14	43.8
Other	4	12.5
Brand/model of CI		
AB	23	71.9
Cochlear	3	9.4
MED-EL	5	15.6
Nurotron	1	3.1
Rehabilitation training after CI		
Yes	30	93.8
No	2	6.3
Bilateral implants		
Simultaneous	9	90.0
In sequence	1	10.0
Genes for deafness		
Yes	18	56.3
No	6	18.8
Unknown	8	25.0
HA wearing before CI		
Yes	21.0	65.6
No	11.0	34.4

SNHL, sensorineural hearing loss; LVAS, large vestibular aqueduct syndrome; HA, hearing aid; CI, cochlear implant.

The participants were further categorized into three subgroups based on the type of CI. We conducted a comparative analysis of the characteristics across the subgroups, and the detailed results are shown in Table 2. The age differences among the subgroups were statistically significant. Children with unilateral implants (12.6 ± 3.7 years) were older than those with bilateral implants (6.4 ± 2.6 years, *p* < 0.001) or those with bimodal stimulation (8.7 ± 3.3 years, *p* = 0.008). The duration from cochlear implantation for children with unilateral implants (119.3 ± 54 months) was also longer than that for those with bilateral implants (50.9 ± 29 months, *p* = 0.001) or bimodal stimulation (69.1 ± 30 months, p = 0.008). However, there were no differences in the age of cochlear implantation or the duration of auditory deprivation among the three subgroups.

**Table 2:** Comparison of the characteristics of Mandarin-speaking pediatric CI recipients with different CI types.

(A)	N	X	S	F	P	*post hoc*
Speech perception accuracy (%)						
Unilateral implants	12	90.1	8.7	0.076	0.927	NA
Bilateral implants	7	91.2	6.6			
Bimodal stimulation	9	89.8	7			
Aided hearing threshold (dBHL)						
Unilateral implants	12	27.8	7.1	3.964	0.031	Unilateral-bilateral: *P* = 0.010
Bilateral implants	8	37	9.4			Unilateral-bimodal: *p* = 0.124
Bimodal stimulation	10	32.7	5.3			Bilateral-bimodal: *p* = 0.224
Age (years)						
Unilateral implants	12	12.6	3.7	10.602	<0.001	Unilateral-bilateral: *P* < 0.001
Bilateral implants	10	6.4	2.6			Unilateral-bimodal: *P* = 0.008
Bimodal stimulation	10	8.7	3.3			Bilateral-bimodal: *p* = 0.122
Time of cochlear implantation (months)						
Unilateral implants	12	119.3	54	8.393	0.001	Unilateral-bilateral: *P* = 0.001
Bilateral implants	10	50.9	29.8			Unilateral-bimodal: *P* = 0.008
Bimodal stimulation	10	69.1	30.5			Bilateral-bimodal: *p* = 0.329
Age of cochlear implantation (years)						
Unilateral implants	12	2.6	1.7	0.335	0.718	NA
Bilateral implants	10	2.1	2.9			
Bimodal stimulation	10	2.8	1.7			
Time of auditory deprivation (months)						
Unilateral implants	12	27.3	20.7	0.523	0.598	NA
Bilateral implants	10	20.4	32			
Bimodal stimulation	10	17.4	14.2			

(B)	Unilateral implants		Bilateral implants		Bimodal stimulation		Chi-square	*p*
	*n*	%	*n*	%	*n*	%		
Gender							1.635	0.442
Men	3	25.0	3.0	30.0	5.0	50.0		
Women	9	75.0	7.0	70.0	5.0	50.0		
Rehabilitation training after cochlear implantation							0.996	0.608
Yes	11	91.7	9	90.0	10	100.0		
No	1	8.3	1	10.0	0	0.0		
The reasons for deafness							4.800	0.308
SNHL+LVAS/Mondini	3	25.0	4	40.0	7	70.0		
SNHL	7	58.3	5	50.0	2	20.0		
Other	2	16.7	1	10.0	1	10.0		
HA wearing before CI							8.727	0.013
Yes	9	75.0	3	30.0	9	90.0		
No	3	25.0	7	70.0	1	10.0		

SNHL, sensorineural hearing loss; LVAS, large vestibular aqueduct syndrome; HA, hearing aid; CI, cochlear implant.

### 3.2 Characteristics of AHT and SPA

The AHT of all pediatric participants was 31.9 ± 8.0 dBHL, and the SPA was 90.3 ± 7.4%. There were no statistically significant differences in SPA among children with unilateral implants, those with bilateral implants, and those with bimodal stimulation. However, the AHT of children with unilateral implants (27.8 ± 7.1 dBHL) was significantly lower than those with bilateral implants (37.0 ± 9.4 dBHL, *p* = 0.010). Furthermore, we conducted a comparative analysis of AHT and SPA between the ears of children receiving bimodal stimulation; the results indicated no discernible difference between the HA and the CI (AHT: 39.3 ± 9.6 dBHL for HAs vs. 36.1 ± 8.8 dBHL for CIs, *p* = 0.465; SPA: 86.2 ± 6.9% for HAs vs. 86.5 ± 9.8% for CIs, *p* = 0.910).

### 3.3 Source localization of the MMN response

**Figure 1:**
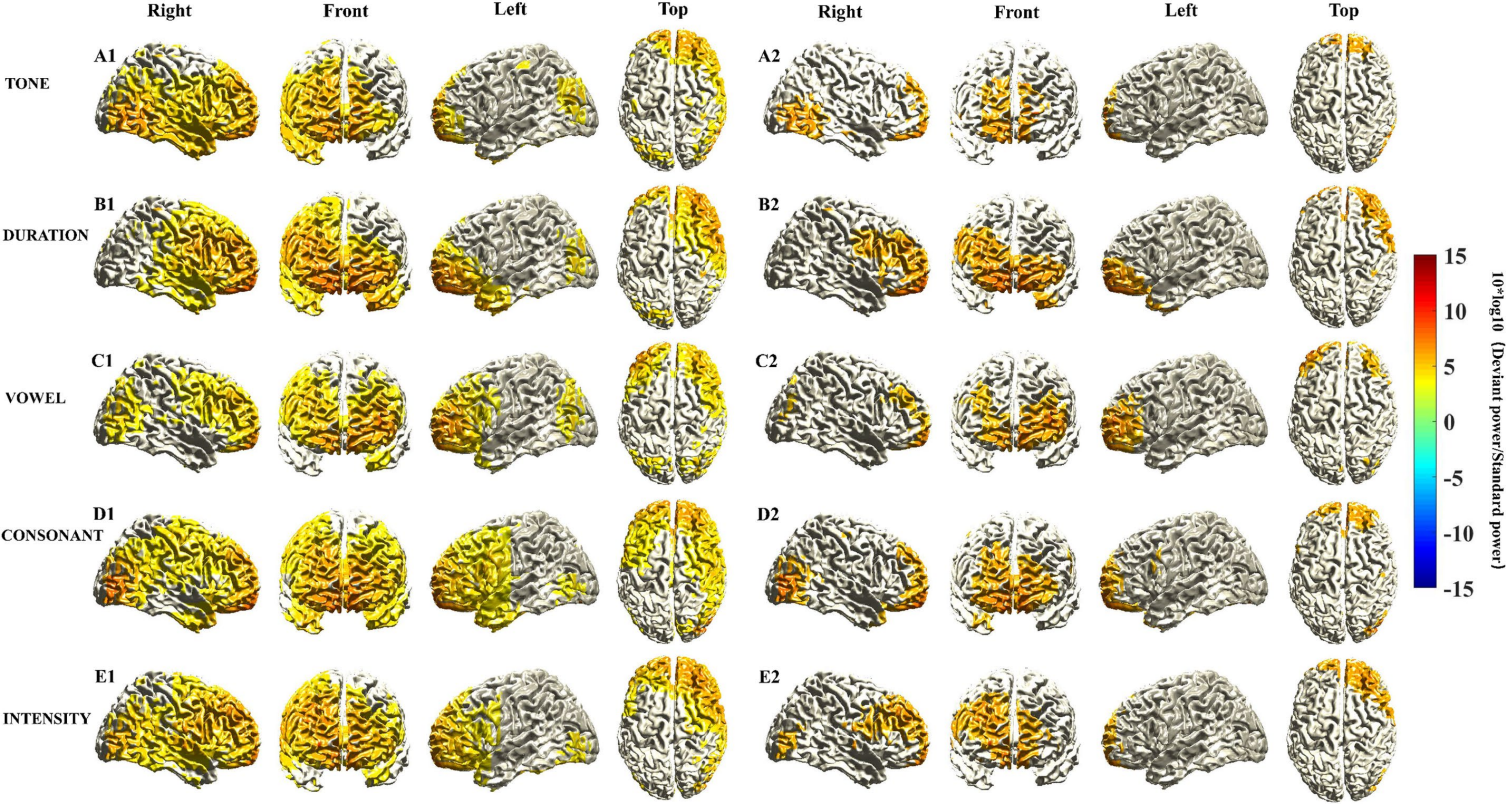
Source localization of MMN responses elicited by five different deviant stimuli. The location of MMN sources was defined as those with a 10 log_10_ (deviant stimuli power/standard stimuli power) value in the top 60% **(A1–E1)** and 35% **(A2–E2)**. The mean sources of the MMN components were calculated using the 20 ms window around the identified timepoint of the trough for each of the different deviant stimuli (see Supplementary Figure S1A).

We identified characteristics of the activated cortical areas of the MMN in response to different Mandarin pronunciation stimuli. First, the activated cortical areas were in the temporal, frontal, parietal, and occipital lobes. Subtle variations in the activation regions and intensities in the cortex were observed in response to different Mandarin pronunciation stimuli. The activation area in the right hemisphere of the brain was more extensive than that in the left hemisphere (Figures 1A1–E1). Second, upon constraining the cortical activation area of the MMN response to the top 35%, the activated cortical areas were predominantly situated in the prefrontal lobe (Figures 1A2–E2). Third, the activation power of MMN responses in each voxel subsequently coalesced into brain regions corresponding to the AAL brain template. Table 3 presents the activation power in various frontal cortex brain regions. Notably, under various Mandarin pronunciation stimuli, the highest activation power of the MMN response was observed in the orbital part, medial and medial orbital region of the superior frontal gyrus.

**Table 3:** The activation power of MMN responses elicited by five different deviant stimuli in various frontal cortex brain regions.

	Tone	Duration	Vowel	Consonant	Intensity
	X ± S	X ± S	X ± S	X ± S	X ± S
SFGdor	4.1 ± 4.5	4.6 ± 3.9	4.5 ± 3.4	4.6 ± 4.1	5.3 ± 4.5
ORBsup	5.2 ± 5.4	4.8 ± 5.6	4.7 ± 4.2	5.1 ± 5.2	5.3 ± 4.6
MFG	3.2 ± 3.7	4.0 ± 3.1	4.5 ± 3.1	4.0 ± 3.5	4.7 ± 3.9
ORBmid	4.7 ± 4.9	4.5 ± 4.8	4.6 ± 4.2	4.6 ± 4.7	5.0 ± 4.5
IFGoperc	2.7 ± 3.5	3.3 ± 3.3	3.3 ± 3.4	3.4 ± 3.1	4.3 ± 3.7
IFGtriang	3.1 ± 3.7	3.6 ± 3.7	4.0 ± 3.9	3.4 ± 3.2	4.6 ± 3.9
ORBinf	3.5 ± 4.7	3.5 ± 4.6	3.1 ± 4.4	3.2 ± 4.5	4.1 ± 4.1
SFGmed	5.0 ± 5.5	4.9 ± 5.4	4.6 ± 5.3	5.8 ± 5.3	6.0 ± 5.1
ORBsupmed	5.8 ± 5.7	5.4 ± 6.4	5.3 ± 4.8	6.0 ± 6.2	6.2 ± 5.2

The activation power of MMN responses was computed using the formula MMN power = 10 log_10_ (deviant stimulus power/standard stimulus power). The mean activation power of MMN responses was computed within a 20 ms time window centered on the identified trough for each type of deviant stimulus (see Supplementary Figure S1A). The activation power of MMN responses in each voxel subsequently coalesced into brain regions corresponding to the AAL brain template. SFGdor, superior frontal gyrus, dorsolateral; ORBsup, superior frontal gyrus, orbital part; MFG, middle frontal gyrus; ORBmid, middle frontal gyrus, orbital part; IFGoperc, inferior frontal gyrus, opercular part; IFGtriang, inferior frontal gyrus, triangular part; ORBinf, inferior frontal gyrus, orbital part; SFGmed, superior frontal gyrus, medial; ORBsupmed, superior frontal gyrus, medial orbital.

### 3.4 Source localization of MMN responses across children with various types of CIs

**Figure 2:**
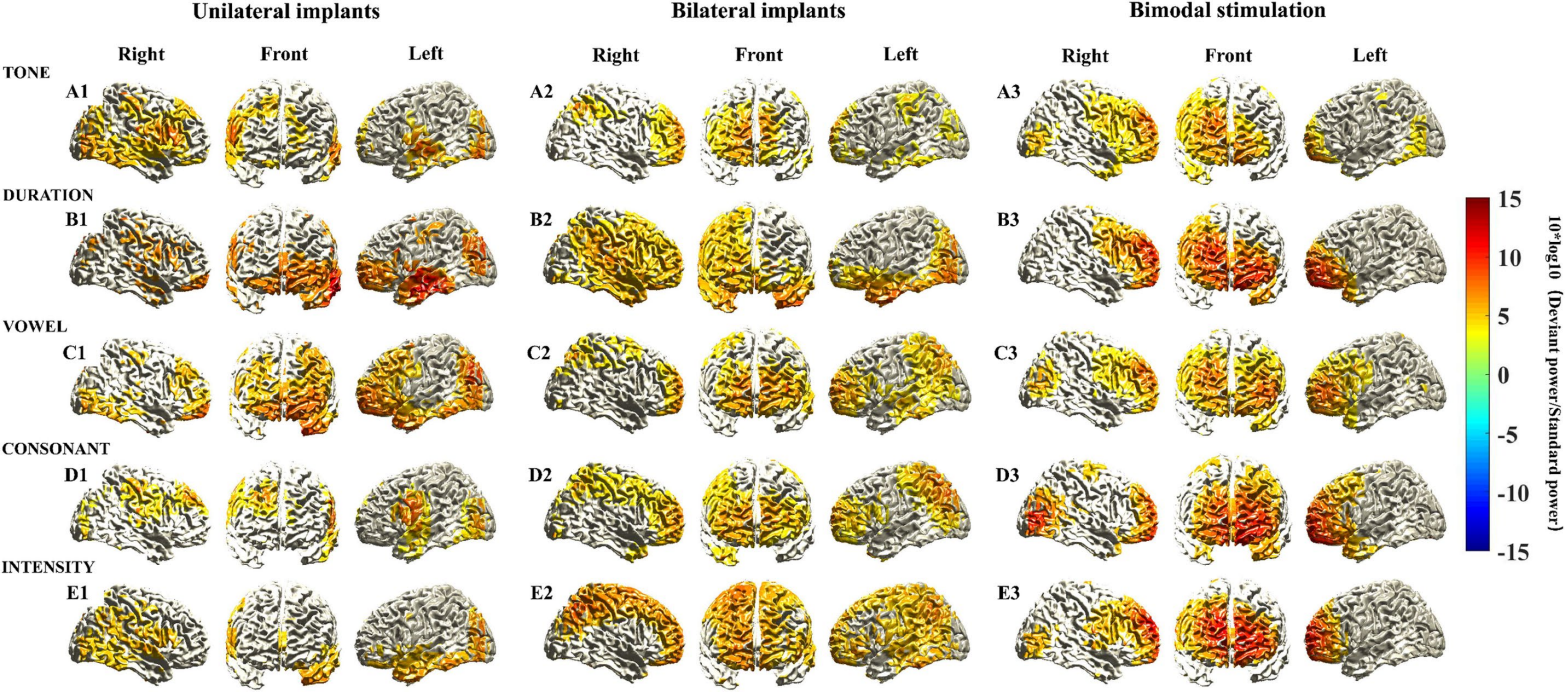
Source localization of MMN responses elicited by five different deviant stimuli among various types of CIs. MMN sources were located as those with a 10 log10 (deviant stimuli power/standard stimuli power) value in the top 60%. The mean sources of the MMN components were calculated using the 20 ms window around the identified timepoint of the trough for each of the different deviant stimuli (see Supplementary Figure S1B).

The participants were further divided into three subgroups, and source localization analysis of MMN responses was conducted within each subgroup. Distinct characteristics of the activated cortical areas in different subgroups were found. Notably, there was pronounced activation in prefrontal cortical areas in children with bimodal stimulation (Figures 2A3–E3). While activated cortical areas in the prefrontal cortex were also evident in children with unilateral and bilateral implants, the strength of activation was relatively diminished. Interestingly, the intensity of the activated cortical areas in the prefrontal cortex did not consistently appear stronger in children with bilateral implants compared to those with unilateral implants under various Mandarin pronunciation stimuli. Specifically, consonant and intensity stimuli presented greater activation strength, whereas duration and vowel stimuli presented weaker activation (Figures 2A1–E1,A2–E2).

The activation power of MMN responses in each voxel subsequently coalesced into brain regions corresponding to the AAL brain template. Table 4 presents the activation power of MMN responses in various frontal cortex brain regions across different types of CIs. Despite the limited sample size reducing the statistical power to detect significant differences in frontal cortex brain regions among different subgroups, several characteristics were still identified. First, the brain regions in the frontal cortical areas of children with bimodal stimulation presented significantly greater activation intensity than did the other two subgroups when exposed to different Mandarin pronunciation stimuli. Second, the heightened activation intensity in the frontal cortical areas of children with unilateral implants, as opposed to those with bilateral implants, was primarily observed in response to tone, duration, and vowel stimuli. Conversely, the increased activation intensity of the brain regions in frontal cortical areas in children with bilateral implants compared with those with unilateral implants were mainly noted in response to consonant and intensity stimuli. Third, the highest activation power of the MMN response to various Mandarin pronunciation stimuli was located primarily in the orbital, medial and medial orbitals of the superior frontal gyrus, and orbital parts of the middle frontal gyrus. The activation characteristics of these four brain regions in the frontal cortex among these three subgroups also conformed to the aforementioned principles.

**Table 4:** The activation power of MMN responses elicited by five different deviant stimuli in various frontal cortex brain regions among various types of CIs.

	Tone		Duration		Vowel		Consonant		Intensity	
	X ± s	*P*	X ± s	*P*	X ± s	*P*	X ± s	*P*	X ± s	*P*
SFGdor		0.663		0.691		0.347		0.321		0.048
Unilateral implants	3.6 ± 5.0		4.6 ± 4.4		4.4 ± 3.4		3.2 ± 5.2		2.5 ± 4.9	
Bilateral implants	2.4 ± 3.7		3.6 ± 3.8		2.5 ± 4.1		2.6 ± 3.7		5.5 ± 3.7	
Bimodal stimulation	4.0 ± 3.9		5.1 ± 3.7		4.7 ± 3.1		5.3 ± 2.8		6.6 ± 1.9	
ORBsup		0.284		0.753		0.649		0.324		0.360
Unilateral implants	4.6 ± 4.8		5.1 ± 5.5		4.2 ± 4.5		2.9 ± 6.1		3.7 ± 4.6	
Bilateral implants	2.3 ± 6.6		3.5 ± 7.0		3.1 ± 5.7		4.3 ± 3.4		5.3 ± 5.1	
Bimodal stimulation	5.9 ± 3.5		5.5 ± 6.2		5.1 ± 3.4		6.3 ± 5.2		6.5 ± 4.1	
MFG		0.676		0.857		0.569		0.699		0.083
Unilateral implants	3.3 ± 4.1		4.0 ± 4.5		4.8 ± 2.9		2.8 ± 4.8		2.5 ± 4.4	
Bilateral implants	2.0 ± 3.0		3.3 ± 3.2		3.4 ± 3.3		3.3 ± 3.8		5.3 ± 3.3	
Bimodal stimulation	2.8 ± 2.1		4.1 ± 2.8		4.1 ± 3.0		4.2 ± 2.2		5.5 ± 1.9	
ORBmid		0.554		0.832		0.797		0.215		0.253
Unilateral implants	3.9 ± 5.5		4.8 ± 4.8		4.1 ± 4.3		2.4 ± 5.8		3.4 ± 4.7	
Bilateral implants	2.9 ± 5.7		3.5 ± 5.8		3.7 ± 5.3		4.5 ± 3.6		5.6 ± 5.2	
Bimodal stimulation	5.4 ± 3.3		4.8 ± 5.7		5.0 ± 3.5		6.0 ± 4.4		6.6 ± 3.5	
IFGoperc		0.290		0.481		0.886		0.993		0.594
Unilateral implants	3.3 ± 3.8		3.4 ± 4.6		3.4 ± 2.9		3.0 ± 4.1		3.2 ± 4.2	
Bilateral implants	1.4 ± 2.3		4.1 ± 3.5		3.3 ± 2.6		2.8 ± 4.4		4.3 ± 2.8	
Bimodal stimulation	1.7 ± 2.2		1.8 ± 4.6		2.8 ± 4.2		2.8 ± 2.3		4.6 ± 2.9	
IFGtriang		0.765		0.882		0.971		0.641		0.320
Unilateral implants	3.0 ± 4.4		3.8 ± 5.5		3.8 ± 3.7		2.2 ± 4.4		3.2 ± 4.5	
Bilateral implants	2.1 ± 2.3		3.3 ± 3.8		3.7 ± 3.4		3.2 ± 4.5		4.7 ± 2.8	
Bimodal stimulation	2.7 ± 1.3		2.8 ± 4.1		4.1 ± 4.1		3.7 ± 2.1		5.5 ± 2.6	
ORBinf		0.690		0.859		0.835		0.230		0.476
Unilateral implants	2.9 ± 5.5		3.8 ± 5.1		2.9 ± 4.3		1.2 ± 5.3		3.3 ± 4.5	
Bilateral implants	2.2 ± 4.5		3.8 ± 5.9		2.1 ± 4.4		2.9 ± 4.2		4.8 ± 3.6	
Bimodal stimulation	3.9 ± 2.5		2.7 ± 4.4		3.3 ± 4.5		4.6 ± 3.8		5.2 ± 3.3	
SFGmed		0.708		0.349		0.384		0.306		0.019
Unilateral implants	4.3 ± 6.0		4.4 ± 5.6		4.0 ± 5.0		4.4 ± 5.9		2.7 ± 4.9	
Bilateral implants	4.0 ± 5.9		3.1 ± 5.4		2.5 ± 6.3		3.3 ± 5.5		5.1 ± 5.6	
Bimodal stimulation	5.9 ± 5.2		6.7 ± 5.3		5.8 ± 4.3		6.9 ± 4.3		8.8 ± 3.1	
ORBsupmed		0.237		0.332		0.417		0.420		0.181
Unilateral implants	5.3 ± 5.1		5.7 ± 6.0		4.6 ± 4.9		3.6 ± 7.0		4.0 ± 4.9	
Bilateral implants	2.9 ± 6.8		2.6 ± 7.8		3.2 ± 6.3		4.8 ± 3.9		5.1 ± 6.0	
Bimodal stimulation	7.1 ± 4.2		7.3 ± 7.2		6.3 ± 3.5		7.1 ± 6.6		8.3 ± 5.3	

The activation power of MMN responses was computed via the formula MMN power = 10 log_10_ (deviant stimulus power/standard stimulus power). The mean activation power of MMN responses was computed within a 20 ms time window centered on the identified trough for each type of deviant stimulus (see Supplementary Figure S1B). The activation power of MMN responses in each voxel subsequently coalesced into brain regions corresponding to the AAL brain template. SFGdor, superior frontal gyrus, dorsolateral; ORBsup, superior frontal gyrus, orbital part; MFG, middle frontal gyrus; ORBmid, middle frontal gyrus, orbital part; IFGoperc, inferior frontal gyrus, opercular part; IFGtriang, inferior frontal gyrus, triangular part; ORBinf, inferior frontal gyrus, orbital part; SFGmed, superior frontal gyrus, medial; ORBsupmed, superior frontal gyrus, medial orbital.

### 3.5 Comparison of source activation across children with various types of CIs

**Figure 3:**
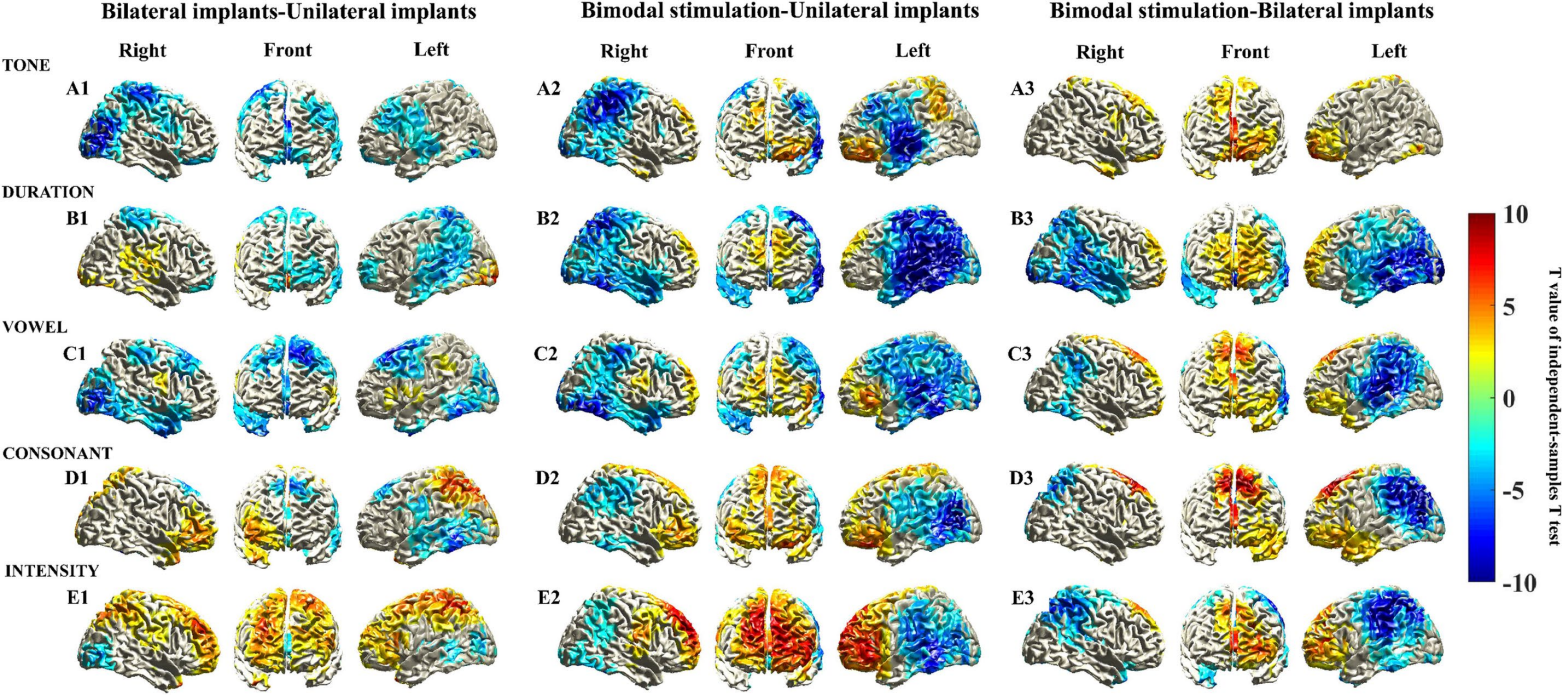
Statistically significant differences in the sources of the MMN responses were elicited by five different deviant stimuli through an independent sample *t* test. Statistically significant differences in the sources of the MMN responses were assessed via a cluster-based random permutation procedure. An independent sample t test was performed to identify the significant voxels, and the t values of these voxels were then interpolated onto the cerebral cortex in this figure. Uncolored cortical regions indicate areas that did not exhibit significant differences (*α* = 5%) in independent sample t tests or that did not survive cluster-based random permutation statistical correction (*α* = 5%, 10,000 substitutions).

To increase the statistical power and ensure the detection of differences in activated cortical regions across different types of CIs, the sample size was increased fourfold through data replication. Consequently, subsequent analysis is based on ideal circumstances. Figure 3 shows the T values of the voxels with significant differences in the cerebral cortex. Compared with children who have unilateral implants, no significant increase in activation intensity was observed in the prefrontal cortical areas of children with bilateral implants when exposed to tone, duration, or vowel stimuli. Cool-toned brain regions (*T* value <0) could be observed in the prefrontal cortex (Figures 3A1–C1). Conversely, the activation intensity of prefrontal cortical brain regions in children with bilateral implants was significantly greater than the intensity in children with unilateral implants when they were exposed to consonant and intensity stimuli. Warm-toned brain regions (T value >0) were observed in the prefrontal cortex (Figures 3D1–E1). The activation intensity of prefrontal cortical brain regions in children with bimodal stimulation was significantly greater than that in children with unilateral implants or bilateral implants when exposed to all Mandarin pronunciation stimuli. Warm-toned brain regions (T value >0) were observed in the prefrontal cortex (Figures 3A2–E2,A3–E3).

We further identified the voxels demonstrating statistical significance to the brain regions in the frontal cortex corresponding to the AAL brain template (Supplementary Table S1). Interestingly, we observed that the brain regions exhibiting the highest activation power of the MMN response, specifically the orbital region, the medial and medial orbital regions of the superior frontal gyrus, and the orbital region of the middle frontal gyrus, were also nearly identical to those brain regions demonstrating statistically significant differences in activation power. Furthermore, these cerebral regions with the statistical significance aligns with those identified in the voxels exhibiting significant differences in the cerebral cortex (shown in Figure 3), suggesting that these brain areas likely reflect variations in cortical activation characteristics among children with different types of CIs.

## 4 Discussions

Our study revealed the activated cortical areas of the MMN response to various Mandarin pronunciation stimuli, with the highest intensity of cortical activation predominantly located in the prefrontal lobe, particularly in the orbital part and medial and medial orbital regions of the superior frontal gyrus. There was pronounced activation in prefrontal cortical areas in children with bimodal stimulation. Activated cortical areas in the prefrontal cortex were also evident in children with unilateral and bilateral implants, but the strength of activation was diminished. The activation of cortical areas did not consistently appear stronger in children with bilateral implants than in those with unilateral implants. Consonant and intensity stimuli exhibited greater strength, whereas duration and vowel stimuli showed weaker activation. Significant differences in frontal cerebral cortex activation among different types of CIs were predominantly observed in the superior frontal gyrus.

By utilizing the Mandarin pronunciation multifeature paradigm in conjunction with a high-density EEG system, this groundbreaking study elucidates the characteristics of activated cortical areas in pediatric CI recipients’ MMN response to various Mandarin pronunciation stimuli. We recognized that techniques such as PET or fMRI would have provided superior MMN localization; however, their applicability to the study of the MMN in CI users, especially in children, is limited because of their invasive nature, significant safety concerns, and lower temporal resolution (Giraud et al., 2001). This study improved the spatial information content of the inverse cortical potential distribution obtained from scalp potentials by increasing the number of electrodes to 256, thereby increasing source imaging and localization accuracy. However, due to ethical and safety considerations, individual brain T1 images obtained from the MRI scans of the CI participants were not processed. Instead, a standard brain model derived from a 10-year-old child’s data was utilized as a substitute; this might have impacted the accuracy of source localization analysis.

We observed that the most pronounced cortical activation of the MMN response was in the prefrontal lobe, specifically within the orbital and medial regions of the superior frontal gyrus. There is compelling evidence indicating that the MMN is not exclusively elicited in the bilateral auditory cortices but originates from prefrontal sources in both hemispheres, particularly during language perception (Rinne et al., 2005; Halgren et al., 2011; Pulvermüller and Shtyrov, 2006). Researchers have reported that children with CIs who demonstrate proficient speech performance show robust frontal cortex activity, suggesting significant involvement of auditory working memory (Schneiders et al., 2012). Conversely, children with CIs who display poor speech performance show heightened temporal cortex activity. Temporal activation during MMN reflects a compensatory strategy, representing an effort to reanalyze the auditory input in the relevant sensory cortex (Ortmann et al., 2013).

We observed subtle variations in the activation regions and intensities in the cortex in response to different Mandarin pronunciation stimuli in pediatric CI recipients. The activation of prefrontal cortical areas by tone deviants showed a slight decrease, whereas no discernible differences were observed in the activation of prefrontal cortical areas by the other four deviants. We previously utilized the same paradigm to elicit MMN responses in individuals with normal hearing. Nevertheless, the activation patterns in the prefrontal cortical regions exhibited distinct characteristics. The prefrontal cortical areas activated by vowel and consonant deviants showed stronger activation, whereas the activities by tone, duration, or intensity deviants were weak or absent (Mao et al., 2024). Numerous studies have provided evidence for the theoretical foundation that differences exist in the processing complexity of various linguistic elements within the auditory cortex (Kiefer et al., 1996). This finding suggests that suprasegmental phonemic stimuli, such as tone, duration, and intensity, may necessitate fewer higher-level auditory cognitive resources for processing than segmental phonemic stimuli, such as vowels and consonants, in individuals with normal hearing (Pakarinen et al., 2007). However, pediatric CI recipients may allocate increased cognitive resources to capture a broader range of auditory cues, compensating for deficiencies in hearing perception due to limitations in auditory perception ability arising from internal neural factors and external equipment constraints. Thus, the prefrontal cortical areas exhibit significant activation in response to tone, duration, or intensity deviants in pediatric CI recipients.

In children with bimodal stimulation, we observed pronounced activation in prefrontal cortical areas, which was more robust than the activation of cortical areas in children with unilateral and bilateral implants. To our surprise, the activation in prefrontal cortical areas did not consistently appear stronger in children with bilateral implants than in those with unilateral implants. This finding contradicts previous evidence indicating that bilateral CIs may confer greater benefits for the central auditory development of pediatric CI recipients (Gordon et al., 2013; Jiwani et al., 2021; Sparreboom et al., 2014). Currently, research on the auditory development of Mandarin-speaking children with various types of CIs predominantly relies on questionnaires and scale assessments (Gao et al., 2021). There are limited neuroimaging evidence-based studies available for reference. To gain a comprehensive understanding of the rehabilitation status of these participants in our study, we also gathered their sociodemographic data, clinical data, postoperative rehabilitation data, and subjective speech audiometry data. Based on the available evidence, we present the following considerations. First, despite an average auditory deprivation period of 17.4 months in children with bimodal stimulation, the presence of residual hearing on the hearing aid side effectively negates the potential period of auditory deprivation, as postulated by theory. We conducted a comparative analysis of the AHT and SPA between the HA-aided ear and the CI-aided ear, and findings indicate no statistically significant difference, suggesting that the HA-aided ear demonstrates sufficient compensatory mechanisms for auditory function. The average duration of cochlear implantation, 69.1 months, also ensures the effective implementation of rehabilitation training for the CI side. Hence, these factors collectively govern the most pronounced activation in prefrontal cortical areas in children with bimodal stimulation. Second, children with unilateral implants were significantly older than those with bilateral implants (12.6 vs. 6.4 years). The duration from cochlear implantation for children with unilateral implants was also significantly longer than that for those with bilateral implants (119.3 vs. 50.9 months). The AHT of children with unilateral implants was significantly lower than that of children with bilateral implants (27.8 vs. 37.0 dB). These evidence indicates that children with unilateral implants undergo more comprehensive auditory training and exhibit superior adaptation to CI devices than those with bilateral implants. Thus, the activation in prefrontal cortical areas in children with unilateral implants was not weaker than that in those with bilateral implants. To some extent, this serves as a compensatory mechanism for the limitations of unilateral hearing. Third, although the activation of cortical areas did not consistently appear stronger in children with bilateral implants compared to those with unilateral implants, children with bilateral implants demonstrated the highest speech perception accuracy at 91.2% and underwent cochlear implantation at the youngest age of 2.1 years among the three subgroups. Therefore, we cannot draw a clear conclusion that there is no advantage to bilateral implants; children with bilateral implants may still exhibit considerable potential for auditory central nervous system development. The superiority of their outcomes over those with unilateral implants will necessitate further follow-up studies and validation.

Under various Mandarin pronunciation stimuli, the highest activation power of the MMN response was predominantly observed in the superior frontal gyrus. Notably, these regions were also found to closely correspond with brain areas showing significant differences in activation power among children with different types of CIs. There is a paucity of direct evidence regarding the impact of superior frontal gyrus activation on auditory function in children with CIs. We conducted a comprehensive literature review to gather potential evidence elucidating the influence of superior frontal gyrus activation on auditory function. First, previous studies have indicated that early hearing deprivation affects not only hearing perception but also higher-level cognitive function (Bigler et al., 2019). The superior frontal gyrus is involved in the regulation of working memory, stress perception, and the modulation of emotional and behavioral control (Koban et al., 2021). We infer that early hearing deprivation, the type of CI implantation, and postoperative rehabilitation training can potentially influence the activation of these brain regions in pediatric CI recipients exhibiting variations in higher-level auditory cognitive functions. Second, the Broca and Wernicke areas play important roles in the production and comprehension of language (Penfield and Jasper, 1954). A recent study revealed a neural tract connecting the Broca area and the superior frontal gyrus, which is hypothesized to be involved in language functions, particularly in speech initiation and spontaneity (Ookawa et al., 2017). Third, research has demonstrated that the human language system involves a complex network of cortical areas working in concert (Fujii et al., 2015; Hickok and Poeppel, 2007). The brain regions located in the superior frontal gyrus play crucial roles as key nodes within the network, and evidence indicates that the superior frontal gyrus exhibits anatomical connectivity with numerous brain regions (Takahashi et al., 2013). In conclusion, activation of the superior frontal gyrus may directly or indirectly impact auditory function in pediatric CI recipients.

Our study has several limitations. First, due to the limited availability of resources for pediatric CI recipients, the study included a relatively small number of participants. The small sample size diminishes the statistical power and undermines the comparability and balance of basic characteristics within each subgroup. To enhance the statistical power, we created an ideal situation by increasing the sample size through a fourfold replication of the data, thereby facilitating more discernible observations of the differences in activated cortical regions among different types of CIs. Simply replicating the data only repeats the existing data pattern and cannot reflect the true variability of the population, which is indeed a limitation of this study. Second, the presence of discharge artifacts from CI partially disrupts normal ERP physiological signals despite our diligent efforts to eliminate these artifacts during the preprocessing of EEG data. Complete avoidance of their interference with normal ERP signals remains challenging. We excluded a significant number of samples with severe artifact interference and were cautious in identifying MMN responses. Third, in this study, we chose the MMN as a neural biomarker to explore the characteristics of auditory cortical activation in pediatric CI recipients. While numerous other ERP components are commonly utilized to evaluate auditory function, reflecting various stages of auditory processing, our objective is to investigate the impact of different types of CIs on these ERP components.

In conclusion, MMN was used as a biomarker in this study to investigate central auditory processing activation patterns during speech perception in Mandarin-speaking pediatric CI recipients with varying device characteristics. In light of these findings, we draw the following conclusions. The prefrontal cortical source array revealed by the MMN in processing higher language functions exhibited significant differences in activation among various types of CIs. It is recommended to opt for bimodal stimulation whenever feasible to optimize auditory benefits while also considering economic factors. For deaf children without residual hearing, bilateral implantation represents the optimal choice. However, careful consideration should be given to surgical indications and the financial circumstances of the family, especially in the absence of supportive policies such as medical insurance, as the procedure is cost-prohibitive for most Chinese families. Unilateral cochlear implantation is not as detrimental as previously perceived for deaf children. Early cochlear implantation, comprehensive auditory training and adaptation to CI devices can optimize the rehabilitation of central auditory processing function in children with CI. To some extent, this serves as a highly efficient compensatory mechanism for the limitations of unilateral hearing.

## Data Availability

The datasets presented in this article are not readily available because the datasets generated and/or analysed during the current study are not publicly available due to our team’s intention to produce additional scholarly publications utilizing the data from this study. Therefore, the database is not accessible to the public at present but can be made available from the corresponding author upon reasonable request. Requests to access the datasets should be directed to Xiang Mao, maxx2003@live.cn.

## Footnotes

^1^ https://www.alivelearn.net/xjview
