# Supplementary material for: Mandarin-speaking children with different types of cochlear implant exhibit variations in the activation patterns of their central auditory processing
Source: Front Neurosci. 2024 Dec 16;18:1520415. doi: 10.3389/fnins.2024.1520415 (PMC11683060; doi:10.3389/fnins.2024.1520415)

**Supplemental Table 1. The prefrontal cortex brain regions with statistically significant differences in activation power according to the independent sample t test.**

| **TONE** | | | | | | | | | |
| --- | --- | --- | --- | --- | --- | --- | --- | --- | --- |
| **Bilateral implants-****Unilateral implants** | | | | | | | | | |
| IFGoperc.L | IFGoperc.R | ORBinf.L | ORBinf.R | IFGtriang.L |  |  |  |  |  |
| IFGtriang.R | **ORBsupmed**.L | **ORBsupmed**.R | MFG.L | **ORBmid**.L |  |  |  |  |  |
| **ORBmid**.R | MFG.R | SFGdor.L | **SFGmed**.L | **SFGmed**.R |  |  |  |  |  |
|  |  | **ORBsup**.L | **ORBsup**.R | SFGdor.R |  |  |  |  |  |
| **Bimodal stimulation-Bilateral implants** | | | | | | | | | |
|  |  |  |  |  | IFGoperc.R | ORBinf.L | ORBinf.R | IFGtriang.L | IFGtriang.R |
|  |  |  |  |  | **ORBsupmed**.L | **ORBsupmed**.R | MFG.L | **ORBmid**.L | **ORBmid**.R |
|  |  |  |  |  | MFG.R | SFGdor.L | **SFGmed**.L | **SFGmed**.R | **ORBsup**.L |
|  |  |  |  |  | **ORBsup**.R | SFGdor.R |  |  |  |
| **Bimodal stimulation-Unilateral implants** | | | | | | | | | |
|  |  |  |  | IFGoperc.L | ORBinf.L | IFGtriang.L | **ORBsupmed**.L | **ORBsupmed**.R | MFG.L |
|  |  |  |  |  | **ORBmid**.L | MFG.R | SFGdor.L | **SFGmed**.L | **SFGmed**.R |
|  |  |  |  |  | **ORBsup**.L | **ORBsup**.R | SFGdor.R |  |  |
| **DURATION** | | | | | | | | | |
| **Bilateral implants-Unilateral implants** | | | | | | | | | |
| SFGdor.R | **ORBsup**.R | **ORBsup**.L | **SFGmed**.R | **SFGmed**.L | IFGoperc.R |  |  |  |  |
| SFGdor.L | MFG.R | **ORBmid**.R | **ORBmid**.L | MFG.L |  |  |  |  |  |
| **ORBsupmed**.R | **ORBsupmed**.L | IFGtriang.R | IFGtriang.L | ORBinf.R |  |  |  |  |  |
|  |  |  |  | ORBinf.L |  |  |  |  |  |
| **Bimodal stimulation-Bilateral implants** | | | | | | | | | |
|  |  | IFGoperc.L | IFGoperc.R | IFGtriang.R | ORBinf.L | ORBinf.R | IFGtriang.L | **ORBsupmed**.L | **ORBsupmed**.R |
|  |  |  |  |  | MFG.L | **ORBmid**.L | **ORBmid**.R | MFG.R | SFGdor.L |
|  |  |  |  |  | **SFGmed**.L | **SFGmed**.R | **ORBsup**.L | **ORBsup**.R | SFGdor.R |
| **Bimodal stimulation-Unilateral implants** | | | | | | | | | |
| IFGoperc.L | IFGoperc.R | ORBinf.L | ORBinf.R | IFGtriang.L | **ORBsupmed**.L | **ORBsupmed**.R | MFG.L | **ORBmid**.L | MFG.R |
|  |  |  |  | IFGtriang.R | SFGdor.L | **SFGmed**.L | **SFGmed**.R | **ORBsup**.L | **ORBsup**.R |
|  |  |  |  |  | SFGdor.R |  |  |  |  |
| **VOWEL** | | | | | | | | | |
| **Bilateral implants-Unilateral implants** | | | | | | | | | |
| ORBinf.R | IFGtriang.R | **ORBsupmed**.L | **ORBsupmed**.R | MFG.L | IFGoperc.L | IFGoperc.R | ORBinf.L | IFGtriang.L |  |
| **ORBmid**.R | MFG.R | SFGdor.L | **SFGmed**.L | **SFGmed**.R |  |  |  |  |  |
|  |  | **ORBsup**.L | **ORBsup**.R | SFGdor.R |  |  |  |  |  |
| **Bimodal stimulation-Bilateral implants** | | | | | | | | | |
|  |  |  |  | IFGoperc.L | IFGoperc.R | ORBinf.L | IFGtriang.L | **ORBsupmed**.L | **ORBsupmed**.R |
|  |  |  |  |  | MFG.L | **ORBmid**.L | MFG.R | SFGdor.L | **SFGmed**.L |
|  |  |  |  |  | **SFGmed**.R | **ORBsup**.L | **ORBsup**.R | SFGdor.R |  |
| **Bimodal stimulation-Unilateral implants** | | | | | | | | | |
|  |  |  | ORBinf.R | IFGtriang.R | IFGoperc.L | IFGoperc.R | ORBinf.L | IFGtriang.L | **ORBsupmed**.L |
|  |  |  |  |  | **ORBsupmed**.R | MFG.L | **ORBmid**.L | **ORBmid**.R | MFG.R |
|  |  |  |  |  | SFGdor.L | **SFGmed**.L | **SFGmed**.R | **ORBsup**.L | **ORBsup**.R |
|  |  |  |  |  | SFGdor.R |  |  |  |  |
| **CONSONANT** | | | | | | | | | |
| **Bilateral implants-Unilateral implants** | | | | | | | | | |
|  |  | IFGoperc.L | IFGtriang.L | **SFGmed**.L | IFGoperc.R | ORBinf.L | ORBinf.R | IFGtriang.R | **ORBsupmed**.L |
|  |  |  |  |  | **ORBsupmed**.R | MFG.L | **ORBmid**.L | **ORBmid**.R | MFG.R |
|  |  |  |  |  | SFGdor.L | **SFGmed**.R | **ORBsup**.L | **ORBsup**.R | SFGdor.R |
| **Bimodal stimulation-Bilateral implants** | | | | | | | | | |
|  |  |  |  |  | IFGoperc.L | IFGoperc.R | ORBinf.L | ORBinf.R | IFGtriang.L |
|  |  |  |  |  | IFGtriang.R | **ORBsupmed**.L | **ORBsupmed**.R | MFG.L | **ORBmid**.L |
|  |  |  |  |  | **ORBmid**.R | MFG.R | SFGdor.L | **SFGmed**.L | **SFGmed**.R |
|  |  |  |  |  | **ORBsup**.L | **ORBsup**.R | SFGdor.R |  |  |
| **Bimodal stimulation-Unilateral implants** | | | | | | | | | |
|  |  |  |  |  | IFGoperc.L | IFGoperc.R | ORBinf.L | ORBinf.R | IFGtriang.L |
|  |  |  |  |  | IFGtriang.R | **ORBsupmed**.L | **ORBsupmed**.R | MFG.L | **ORBmid**.L |
|  |  |  |  |  | **ORBmid**.R | MFG.R | SFGdor.L | **SFGmed**.L | **SFGmed**.R |
|  |  |  |  |  | **ORBsup**.L | **ORBsup**.R | SFGdor.R |  |  |
| **INTENSITY** | | | | | | | | | |
| **Bilateral implants-Unilateral implants** | | | | | | | | | |
|  |  |  |  |  | IFGoperc.L | IFGoperc.R | ORBinf.L | ORBinf.R | IFGtriang.L |
|  |  |  |  |  | IFGtriang.R | **ORBsupmed**.L | **ORBsupmed**.R | MFG.L | **ORBmid**.L |
|  |  |  |  |  | **ORBmid**.R | MFG.R | SFGdor.L | **SFGmed**.L | **SFGmed**.R |
|  |  |  |  |  | **ORBsup**.L | **ORBsup**.R | SFGdor.R |  |  |
| **Bimodal stimulation-Bilateral implants** | | | | | | | | | |
|  |  | ORBinf.R | IFGtriang.R | **ORBmid**.R | IFGoperc.L | IFGoperc.R | ORBinf.L | IFGtriang.L | **ORBsupmed**.L |
|  |  |  |  |  | **ORBsupmed**.R | MFG.L | **ORBmid**.L | MFG.R | SFGdor.L |
|  |  |  |  |  | **SFGmed**.L | **SFGmed**.R | **ORBsup**.L | **ORBsup**.R | SFGdor.R |
| **Bimodal stimulation-Unilateral implants** | | | | | | | | | |
|  |  |  |  |  | IFGoperc.L | IFGoperc.R | ORBinf.L | ORBinf.R | IFGtriang.L |
|  |  |  |  |  | IFGtriang.R | **ORBsupmed**.L | **ORBsupmed**.R | MFG.L | **ORBmid**.L |
|  |  |  |  |  | **ORBmid**.R | MFG.R | SFGdor.L | **SFGmed**.L | **SFGmed**.R |
|  |  |  |  |  | **ORBsup**.L | **ORBsup**.R | SFGdor.R |  |  |

The frontal cortex brain regions with statistically significant differences in activation power across the sources of the MMN responses were assessed via a cluster-based random permutation procedure. Independent sample t tests (α = 5%) and cluster-based random permutation statistical correction (α = 5%, 10000 substitutions) were performed to identify the significant voxels. The xjView toolbox was utilized to identify voxels demonstrating statistical significance to the cortex regions. This section focuses exclusively on the prefrontal cortex regions exhibiting differential activation patterns. The brain regions of the prefrontal cortex with negative T values are presented on the left, and those with positive T values are presented on the right. The orbital part, medial and medial orbital parts of the superior frontal gyrus, and orbital part of the middle frontal gyrus are highlighted in bold since these brain regions presented the highest activation power in the MMN response.

SFGdor. L/R, superior frontal gyrus, dorsolateral; ORBsup. L/R, superior frontal gyrus, orbital part; MFG. L/R, middle frontal gyrus; ORBmid. L/R, middle frontal gyrus, orbital part; IFGoperc. L/R, inferior frontal gyrus, opercular part; IFGtriang. L/R, inferior frontal gyrus, triangular part; ORBinf. L/R, inferior frontal gyrus, orbital part; SFGmed. L/R, superior frontal gyrus, medial; ORBsupmed. L/R, superior frontal gyrus, medial orbital.

**Supplement Figure 1a. Time domain analysis of MMN responses elicited by five different deviant stimuli.**


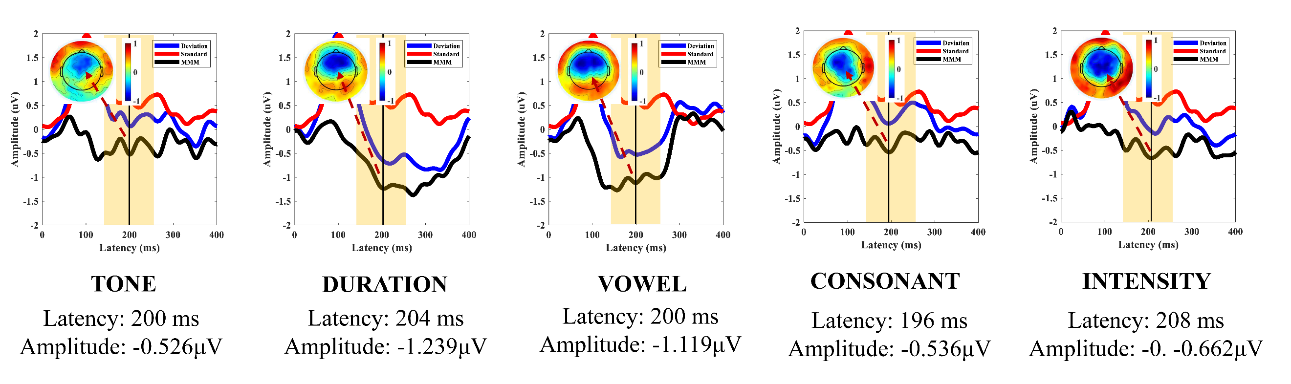


**Supplement Figure 1b. Time domain analysis of MMN responses elicited by five different deviant stimuli among various types of CIs.**


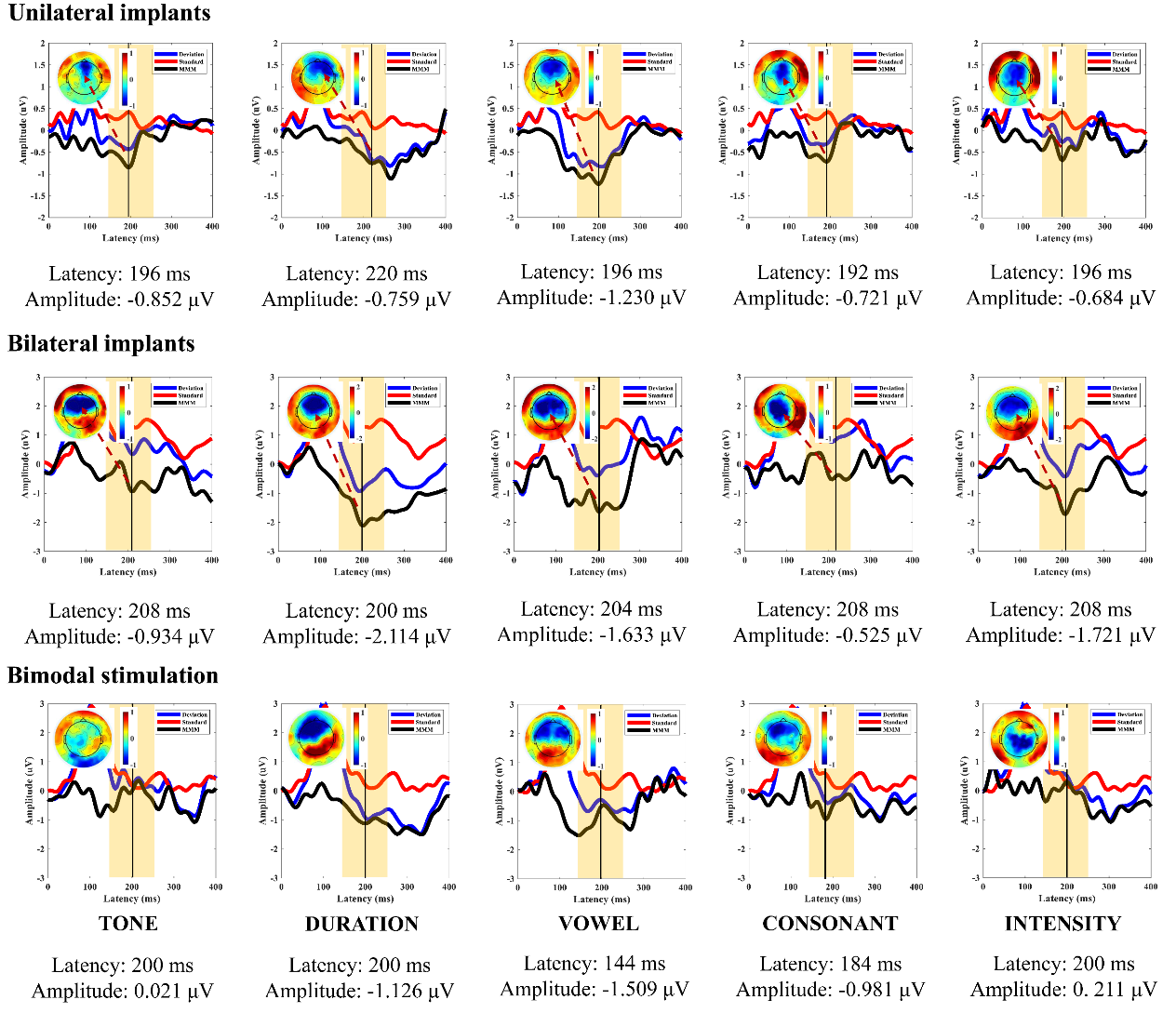

Supplement: Supplementary file 1 [file Data_Sheet_1.docx]
